# A comparative gene co-expression analysis using self-organizing maps on two congener filmy ferns identifies specific desiccation tolerance mechanisms associated to their microhabitat preference

**DOI:** 10.1186/s12870-019-2182-3

**Published:** 2020-02-04

**Authors:** Enrique Ostria-Gallardo, Giovanni Larama, Graciela Berríos, Ana Fallard, Ana Gutiérrez-Moraga, Ingo Ensminger, León A. Bravo

**Affiliations:** 1 Laboratorio de Fisiología Vegetal, Centro de Estudios Avanzados en Zonas Áridas CEAZA, La Serena, Chile; 2 Scientific and Technological Bioresource Nucleus, Universidad de La Frontera, Temuco, Chile; 3 Centro de Excelencia de Modelación y Computación Científica, Facultad de Ingeniería y Ciencias, Universidad de La Frontera, Temuco, Chile; 4 Universidad Autónoma de Chile, Santiago, Chile; 5 Department of Biology, University of Toronto, Toronto, ON Canada; 6 Laboratorio de Fisiología y Biología Molecular Vegetal, Departamento de Cs. Agronómicas y Recursos Naturales, Facultad de Cs. Agropecuarias y Forestales, Instituto de Agroindustria, Universidad de La Frontera, Temuco, Chile

**Keywords:** Hymenophyllaceae, Poikilohydric, Homoiochlorophyllous, Temperate rainforest, RNA-seq transcriptome, Neural network, Gene co-expression

## Abstract

**Background:**

Filmy-ferns (Hymenophyllaceae) are poikilohydric, homoiochlorophyllous desiccation-tolerant (DT) epiphytes. They can colonize lower and upper canopy environments of humid forest. Filmy-ferns desiccate rapidly (hours), contrasting with DT angiosperms (days/weeks). It has been proposed that desiccation tolerance in filmy-ferns would be associated mainly with constitutive features rather than induced responses during dehydration. However, we hypothesize that the inter-specific differences in vertical distribution would be associated with different dynamics of gene expression within the dehydration or rehydration phases. A comparative transcriptomic analysis with an artificial neural network was done on *Hymenophyllum caudiculatum* (restricted to lower canopy) and *Hymenophyllum dentatum* (reach upper canopy) during a desiccation/rehydration cycle.

**Results:**

Raw reads were assembled into 69,599 transcripts for *H. dentatum* and 34,726 transcripts for *H. caudiculatum*. Few transcripts showed significant changes in differential expression (DE). *H. caudiculatum* had ca. twice DE genes than *H. dentatum* and higher proportion of increased-and-decreased abundance of genes occurs during dehydration. In contrast, the abundance of genes in *H. dentatum* decreased significantly when transitioning from dehydration to rehydration. According to the artificial neural network results, *H. caudiculatum* enhanced osmotic responses and phenylpropanoid related pathways, whilst *H. dentatum* enhanced its defense system responses and protection against high light stress.

**Conclusions:**

Our findings provide a deeper understanding of the mechanisms underlying the desiccation tolerance responses of two filmy ferns and the relationship between the species-specific response and the microhabitats these ferns occupy in nature.

## Background

Plant evolution has shaped several acclimation mechanisms and adaptations to withstand short and long-term periods of water deficit. However, most plants cannot survive desiccation, except for a small group of so-called resurrection plants [1, 2]. Desiccation tolerance is a complex trait that involves the coordination of a cascade of molecular events divided into constitutive and inducible mechanisms to protect/repair cells and tissues against oxidative damage, and disruptions of metabolism and cell ultrastructure [2, 3]. In general, the physiological and metabolic components of desiccation tolerance in resurrection plants resemble a combination of processes underlying drought stress responses and seed maturation [4, 5]; however, the molecular “switches” and regulatory pathways behind desiccation tolerance are largely unknown [6]. Resurrection plants are classified into two groups according to their sensitivity, mechanisms of responses, and the velocity of water loss [7]. Those that can survive rapid water loss, such as mosses, possess constitutive (pre-existing) morpho-physiological desiccation tolerance mechanisms employing cellular repair mechanisms induced after rehydration, in contrast to those that survive only if water loss is gradual, which rely upon cellular protection mechanisms induced during dehydration.

Surprisingly, resurrection plants are also found in humid environments such as tropical and temperate rain forests [8]. One example is *Lindernia brevidens*, an angiosperm species from tropical rain forests [9]. The epiphytic ferns of the Hymenophyllaceae family (Pteridophyta) are another group of resurrection plants living in humid ecosystems [8]. The members of Hymenophyllaceae are called filmy ferns because they possess membranous fronds of a single layer of cells, normally lack cuticles, with undifferentiated epidermis, and lack of stomata [10, 11]. Because the features of their leaves and the recurrent exposure to rapid desiccation-rehydration events, these ferns evolved a poikilohydric and homoiochlorophyllous strategy, most typical of bryophytes, showing a high desiccation tolerance and the capacity to rapidly rehydrate [10, 12, 13].

**Fig. 1:**
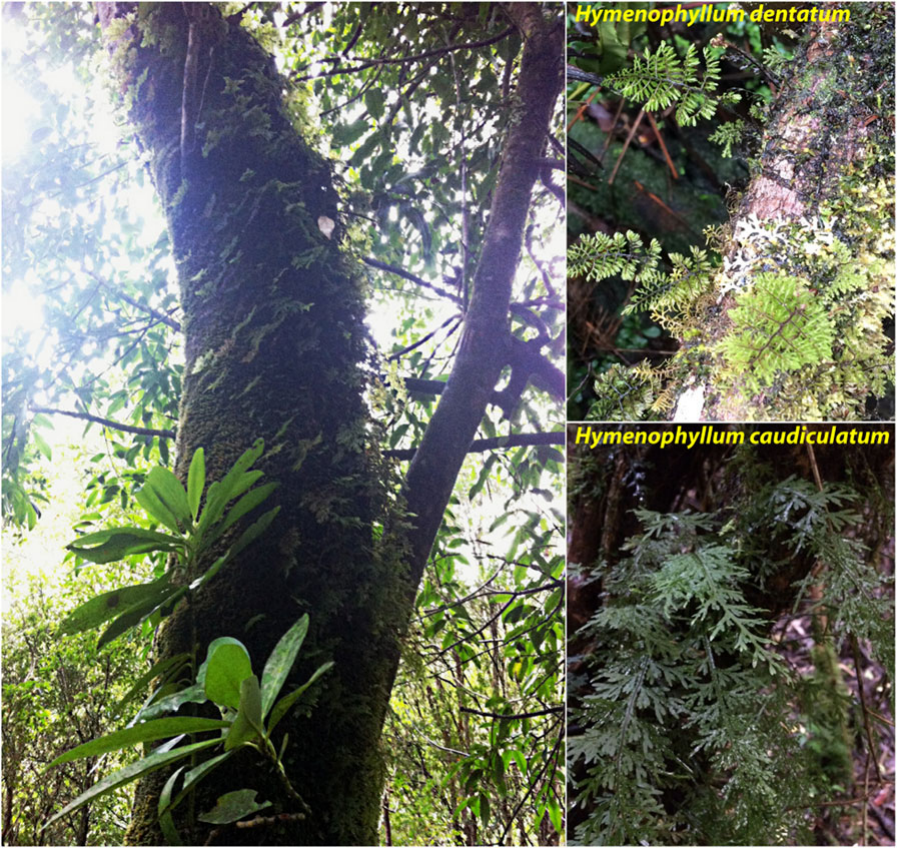
Filmy ferns in their natural environment. At the left of the image a reference of a host tree covered with a carpet of epiphytic filmy ferns. At the right, the two species of resurrection filmy ferns studied, attached to the trunk of their host tree

The Hymenophyllaceae family is represented by 16 endemic species to the Chilean temperate rain forest. They are an important component of the epiphytic species diversity. Filmy ferns can colonize mostly all vertical strata of their host tree, although intraspecific habitat requirements have been reported [11, 14, 15]. For example, *Hymenophyllum caudiculatum* and *Hymenophyllum pectinatum* are restricted to the lower portion of the host trunk (from the ground to 1 m of vertical height), where light availability is very low (10–100 μmol photons m^− 2^ s^− 1^), and humidity is high. Other species (e.g., *H. dentatum*, *H. plicatum*) extend their vertical distribution to heights above 10 m (were PDF eventually reach ≥1000 μmol photons m^− 2^ s^− 1^) [11, 13, 15]. Both light intensity (PFD) and vapor pressure deficit (VPD) increase with the height of the host plant, whereas relative humidity decreases. Therefore, a species that reaches the top of trunks is prone to suffer frequent desiccation and photoinhibition. The wide spectrum of habitats, from very sheltered, high steady humidity environments to higher light, lower humidity along the vertical distribution over host trees offers a unique opportunity to address questions about how molecular and physiological mechanisms are shaped in congeneric populations differing in microhabitat preferences and ability to tolerate desiccation. Questions such as: are the molecular mechanisms responsible for protection/repair of these filmy ferns against tissue desiccation inducible during dehydration? or constitutive (pre-existing), such as those exhibited by rapidly desiccating bryophytes? Is the dynamic of gene expression similar between filmy fern species from lower light, higher humidity environments with species from higher light, lower humidity environments? Do these filmy ferns invoke similar gene functions during a cycle of desiccation-rehydration? As filmy ferns are frequently and rapidly dehydrated, it has been proposed that desiccation tolerance in these ferns would be associated mainly with constitutive features rather than induced responses during dehydration. However, we hypothesize that the inter-specific differences in vertical distribution would be associated with different dynamics of gene expression within the dehydration or rehydration phases. Published data partially support this idea [16, 17]. Advances in Next Generation Sequencing (NGS) tools have brought important advantages to uncover underlying mechanisms that control plant responses associated with natural constraints explored *in situ* or under experimental conditions. Here we used RNA-seq on the Illumina Hi-seq platform to study and compare the transcriptional responses of *H. caudiculatum* and *H. dentatum*, two Hymenophyllaceae species with contrasting vertical microhabitat preferences along host trees and different rates of water loss (Fig. 1). Specifically, we examined at the dynamics of gene expression and a Weighted Gene Co-expression Network (WGCNA) [18] coupled with neural artificial networks [19–21] of fronds subjected to experimental desiccation-rehydration cycles to identify commonalities and differences on gene expression dynamics associated to its water status underlying their resurrection strategy.

## Results

### Changes in ferns’ relative water content and maximum quantum efficiency during desiccation-rehydration cycle

**Fig. 2:**
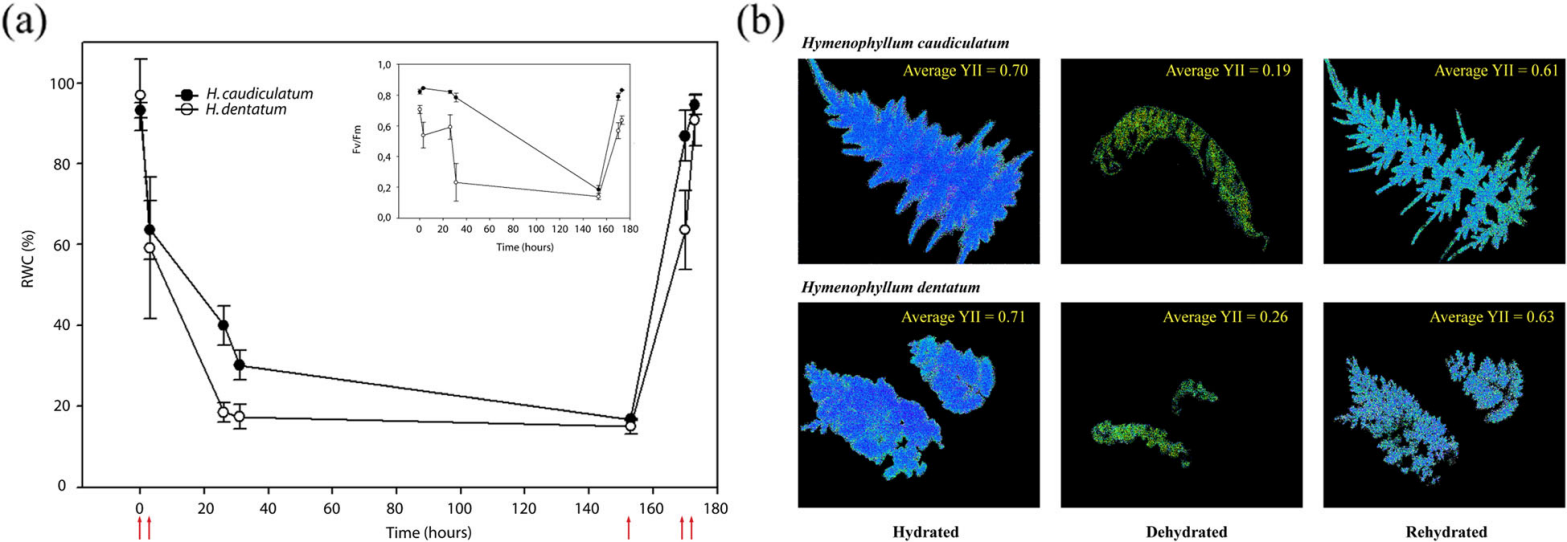
Characterization of desiccation-rehydration cycle in *H. caudiculatum* and *H. dentatum*. **a** Changes in relative water content (RWC) and photochemical efficiency parameter (Fv/Fm, inserted panel) of *H. caudiculatum* and *H. dentatum* during a dehydration and rehydration process. Red arrows indicate sampling of fronds during the dehydration-rehydration process used for the RNAseq library construction. **b** Average Y(II) images measured on dark adapted detached frond of *H. caudiculatum* and *H. dentatum* at full hydration, dehydrated, and rehydrated states

The two filmy fern species showed a similar rate of dehydration during the first three hours after cessation of irrigation (beginning of dehydration), both reaching ca. 60% of relative water content (RWC) (Fig. 2a). From 3 to 25 h without irrigation, *H. dentatum* losses water faster than *H. caudiculatum*, reaching 18 and 30% RWC respectively. During this period of dehydration, the maximum quantum efficiency (Fv/Fm) drastically decayed from about 0.7 to 0.2 in *H. dentatum* but remained nearly 0.78 in *H. caudiculatum* (Fig. 2a insert). After a week without irrigation, both reached a RWC between 11 to 17% and a Fv/Fm near to 0.2 (Fig. 2a). When irrigation was reestablished, *H. caudiculatum* had a faster rehydration and recovery of Fv/Fm compared to *H. dentatum*. Nevertheless, both species reached similar maximum photochemical efficiency (Fv/Fm) by the end of the experiment. Recovery of photochemical efficiency at the whole frond level was confirmed by imaging fluorescence (Fig. 2b).

### Transcriptional profile and transcripts annotation

A total of 111,495,169 and 110,988,488 paired-end reads (101 bp) were obtained after sequencing libraries of *H. caudiculatum* and *H. dentatum*, on the Illumina HiSeq2000 platform (Additional file 1: Table S1). Following the removal of low-quality reads and duplicated reads, we performed a *de novo* transcriptome assemblies with Trinity software by using a set of ~ 85 million reads for *H. caudiculatum* and ~ 87 million reads for *H. dentatum* (Additional file 1: Table S1). The initial assemblies resulted in 161,689 contigs for *H. caudiculatum* and 332,003 contigs for *H. dentatum*, which were refined to remove low supported transcripts. Transcripts with an estimated abundance lower than 1 FPKM and highly similar or redundant transcripts with a sequence similarity higher than 95% were removed. The resulting transcriptomes are represented by 34,726 contigs for *H. caudiculatum* and 69,599 contigs for *H. dentatum* (Additional file 1: Table S2). Although the number of transcripts decreased significantly during the refinement, ca. 80 and 70% of high-quality reads were mapped to the *H. caudiculatum* and *H. dentatum* transcriptomes, respectively.

**Fig. 3:**
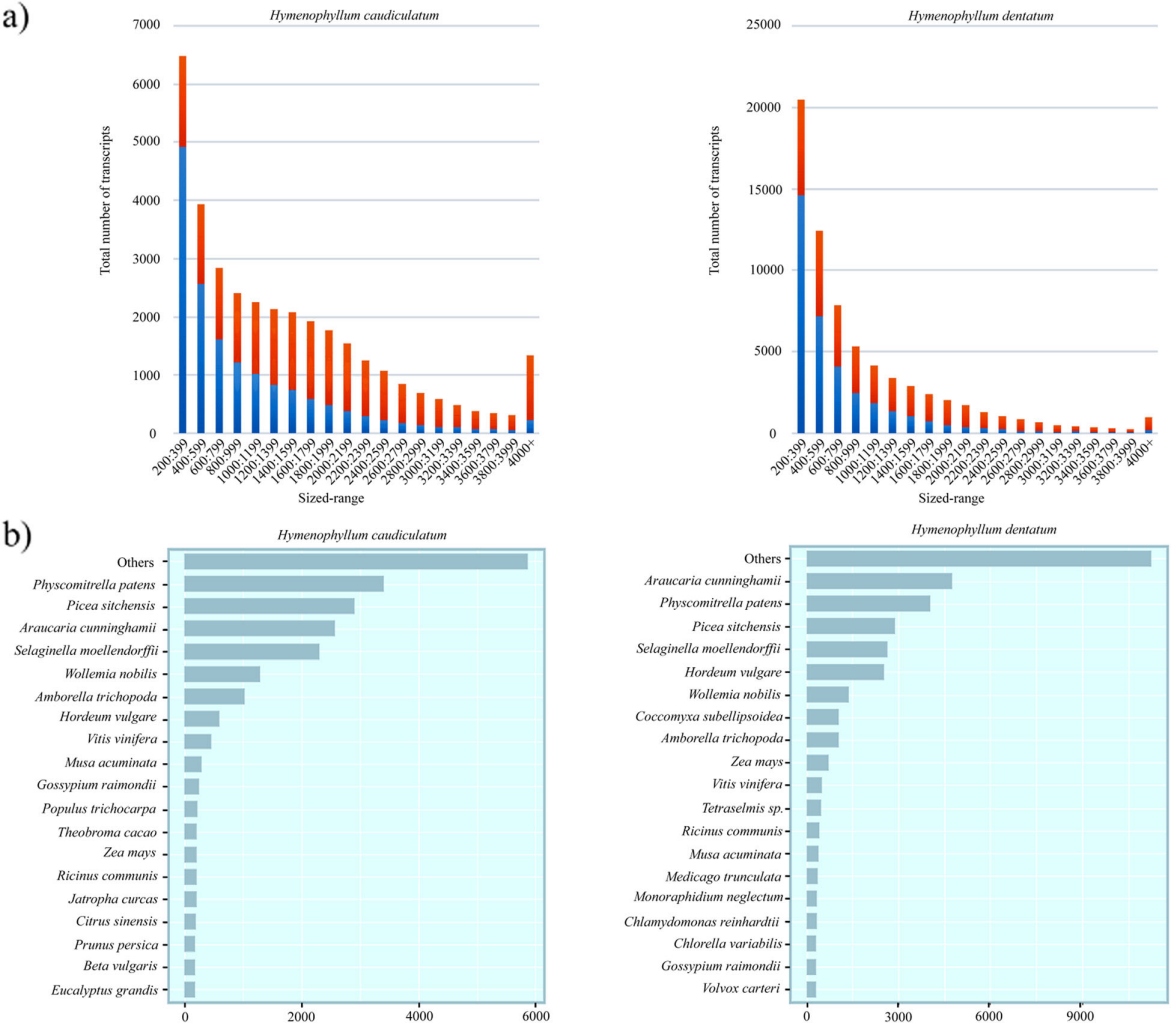
**a** Transcript size distribution showing the proportion those sequences with blast hits (blue) and without blast hits (red) in the final transcriptome assemblies. **b** Top-hit species distribution of the final transcriptome of *H. caudiculatum* and *H. dentatum* showing abundance of top hits to sequences of Bryophyta, Lycophyta, and Pinophyta

The final transcriptome assemblies were aligned to the SwissProt database for annotation, with an alignment rate of ca. 50% for the transcripts of each transcriptome. In spite of the low identification rate, most of the unknown transcripts (~ 80% in *H. dentatum*, and ~ 65% *H. caudiculatum*; Additional file 1: Table S3) belonged to small size transcripts (< 1000 bp; see Fig. 3a). An insight into the taxonomic distribution of top blast hits of transcripts revealed that both *H. caudiculatum* and *H. dentatum* had among their top hits a high amount of sequences belonging to the model moss *Physcomitrella patens* and the lycophyte *Sellaginella moellendorffii* (Fig. 3b), which is consistent with their poikilohydry strategy and the regressive evolution hypothesis [22].

### Differential expression analysis and functional annotation

**Fig. 4:**
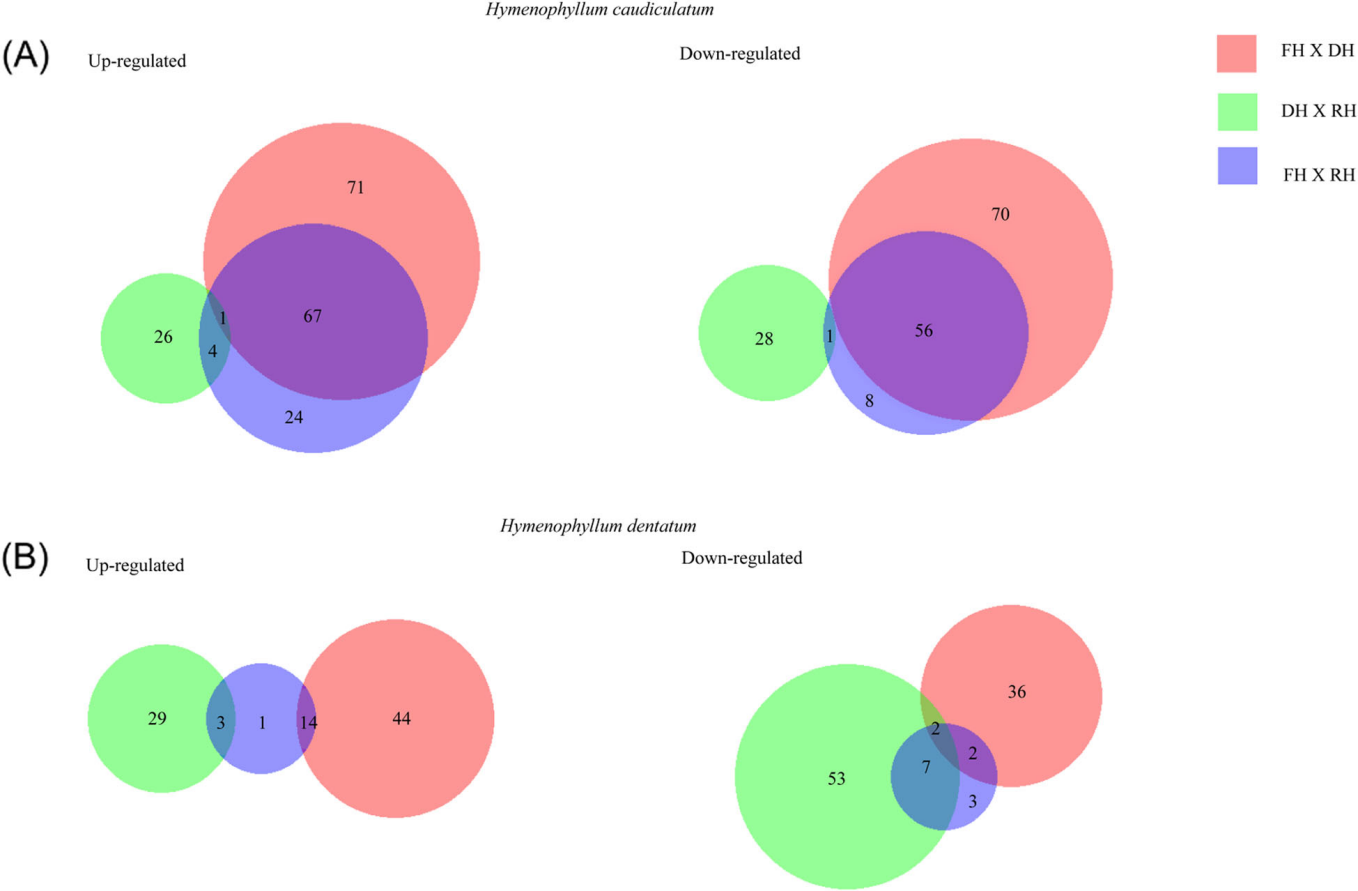
Venn diagrams showing up-and-down regulated genes in *H. caudiculatum* (**a**) and *H. dentatum* (**b**). The size of each circle is proportional to the number of unique, annotated, and non-duplicated up-or-down regulated genes for each of the species. Numbers inside the circles indicate the number of genes differentially expressed by each pair-wise comparison among the hydration states of the fronds (FH, full hydrated; DH, dehydrated; RH, rehydrated). In *H. caudiculatum*, the comparison between FH X DH contributes the largest number of differentially expressed genes (up-and-down regulated). For *H. dentatum*, the comparison between FH X DH contributes the highest number of up-regulated genes, whereas DH X RH contributes the highest number of down-regulated genes. Venn diagrams were generated by using BioVenn (Hulsen et al., 2008)

After quality filtering and refinement, we examined the expression dynamics of annotated genes during the desiccation-rehydration cycle by pairwise comparisons by using a fold change ≥2 and a FDR < 0.05 as cut-off (Additional file 2: Dataset S1 and S2). In both species, few transcripts showed significant changes in differential expression (DE). For *H. caudiculatum*, the highest number of DE genes occurred during the dehydration process with a total of 265 DE genes, where most of them [139] showed an increase in their abundance (Fig. 4). In *H. dentatum*, the number of DE genes that increase and decrease their abundance were similar. However, among the different hydration states, the abundance of genes decreases significantly when transitioning from dehydration to rehydration. When comparing both species, *H. caudiculatum* presents ca. twice DE genes of *H. dentatum* and a higher proportion of both, increase and decrease abundance of genes under the dehydration process (Fig. 4).

**Fig. 5:**
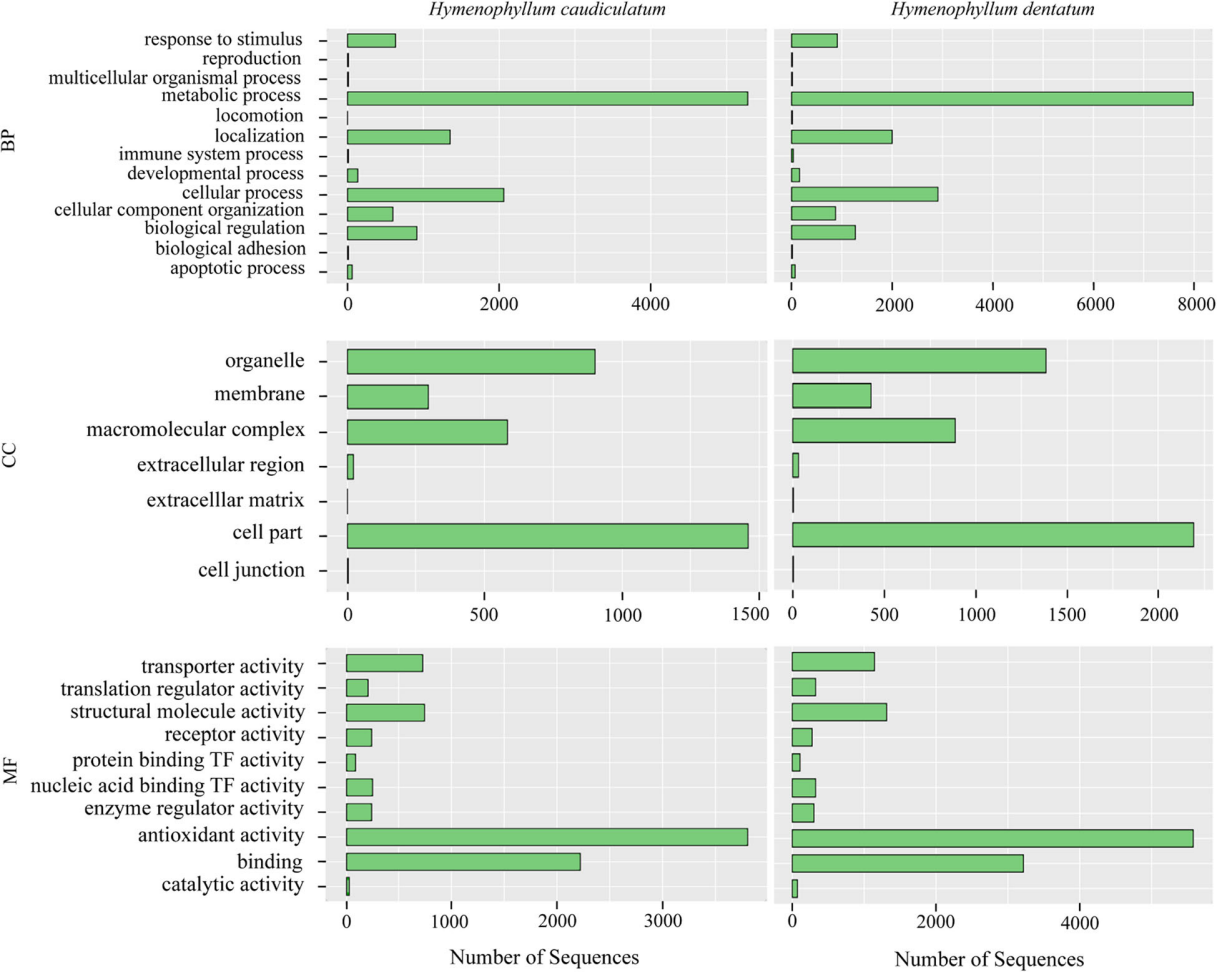
GO-category distribution of annotated genes for *H. caudiculatum* and *H. dentatum* among level 2 GO categories: biological process (BP), cellular component (CC), and molecular function (MF)

From the differentially expressed transcripts we explored the function of the gene products by conducting a Gene Ontology analysis (GO) (see Material and Method for details). Both species showed similar enrichment pattern of sequences for each GO category (Fig. 5). For example, at the biological process category (BP), there was a high number of sequences in the metabolic process (> 4000 sequences) and in cellular process (> 2000 sequences). In the cellular component category (CC), organelle and cell part components showed the highest enrichment of sequences (e.g., ca. 1000 and 1500 sequences for organelle, for *H. dentatum* and *H. caudiculatum*, respectively). Finally, in the molecular function category (MF), the highest accumulation of sequences was found to be in the antioxidant activity, followed by binding process (Fig. 5).

In parallel to the differential expression analysis, we also observed all those highly abundant transcripts based on the FPKM value but with a fold change vale < 2, i.e., constitutive highly abundant genes without significant change along the dehydration-rehydration cycle. A total of 102 transcripts for *H. caudiculatum*, and 128 for *H. dentatum* show that there are broadly three main processes involved in the desiccation tolerance of these two filmy ferns, namely translation, photosynthesis and antioxidant activity (See Additional file 2: Dataset S3 for the detailed list of the highly abundant constitutive transcripts in each species).

### Transcripts clustering patterns and gene co-expression network of *H. caudiculatum* and *H. dentatum* across dehydration-rehydration cycle

**Fig. 6:**
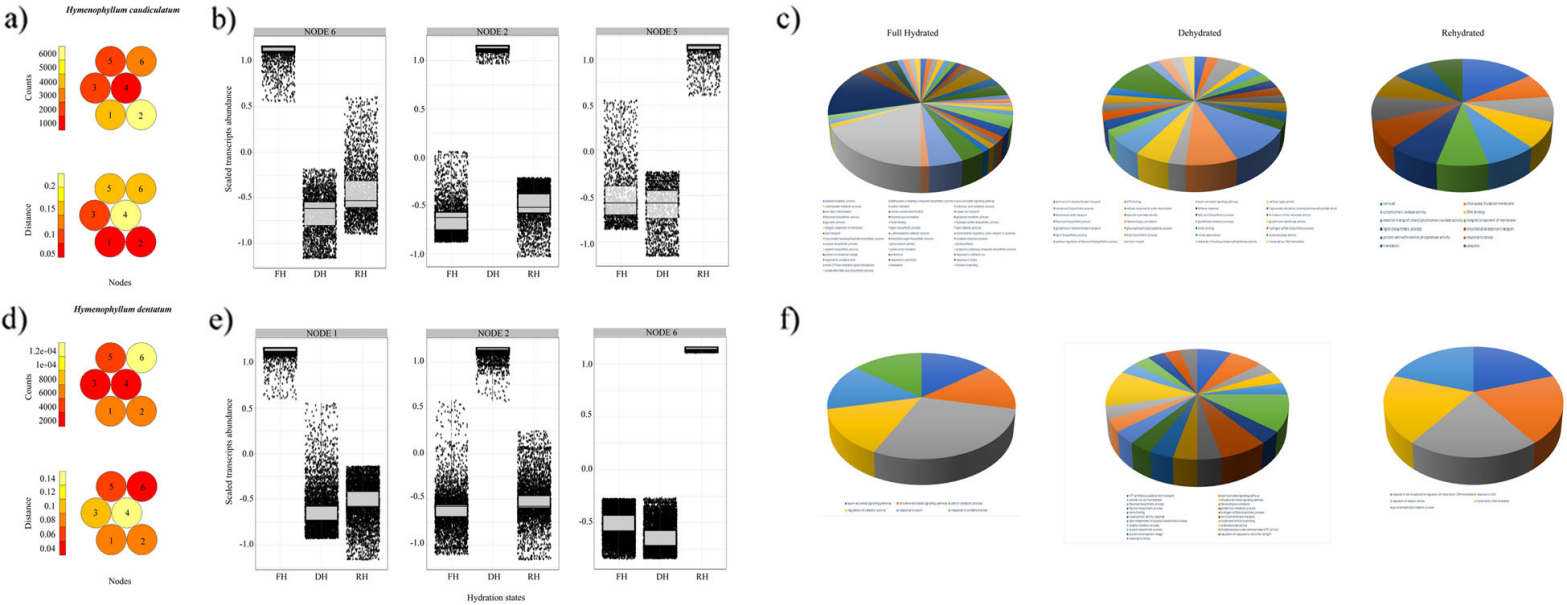
Mapping quality, clustering results for self-organizing maps (SOM) and GO enrichment of transcripts with similar and significant accumulation patterns associated to a given frond hydration state in *H. caudiculatum* (**a-c**) and *H. dentatum* (**d-f**). The heatmaps (**a** and **d**) shows the number of genes (counts) assigned to each SOM Node (numbered from 1 to 6) and the mean Euclidean distance of genes (distance) mapped to the particular Nodes. The constructed maps with a 2 × 3 hexagonal topology shows a reasonable spread out and small distances over the maps, indicating a good mapping process. Nodes with similar codebook vectors (i.e., the patterns of gene neighboring at each hydration state) lie closer to each other. Red color indicates low count and distance, whereas light yellow indicates high count and distance. From the total six Nodes defined after SOM, boxplots were used to visualize and select those Nodes describing the highest pattern of differentially expressed genes for a given hydration state in *H. caudiculatum* (**b**) and *H. dentatum* (**e**) (see details in the Result section). For each boxplot, horizontal line represents the median, and bars represent the maximum and minimum values of the scaled gene abundance. The pie charts indicate the GO enrichment terms for the annotated transcripts with the highest pattern of differential expression for each hydration state in *H. caudiculatum* (**c**) and *H. dentatum* (**f**). X axis labels in boxplots read as follow. FH = Full Hydration; DH = Dehydration; and RH = Rehydration

From the annotated DE genes, we studied the dynamics of gene expression in these two resurrection filmy ferns to identify, firstly, transcripts with similar accumulation patterns in response to a given hydration status, and secondly, the complexity of the interaction and co-expression of genes related with their desiccation and rehydration responses. The self-organizing maps (SOM) partitioned the DE genes into six clusters (hereafter nodes) arranged as a map (Fig. 6 a-c). The underlying topology of the SOM shows distinct accumulation patterns of genes membership across nodes (Fig. 6 a-c), and prominent densities of transcripts for a given hydration state within a node (Fig. 6 b-d), which in topological terms, reflects similar accumulation patterns. Thus, SOM Node 6 in *H. caudiculatum* and SOM Node 1 in *H. dentatum* have a prominent density of transcripts associated with the full hydrated state (FH). For the dehydrated and re-hydrated conditions, respectively, the enrichment of transcripts was observed in Nodes 3 and 2 in *H. caudiculatum* (Fig. 6b), and Nodes 2 and 6 in *H. dentatum* (Fig. 6d). In order to ascertain if SOM-based clustering yields biologically relevant information, we determined GO enrichment in those nodes. Specifically, we found that for *H. caudiculatum*, transcripts of the dehydrated state (Node 3) were enriched in functional categories related to stress signaling and response, photosynthesis and photosystem II stabilization and repair, unsaturation of fatty acid, and lignin biosynthetic process. Next, transcripts in the rehydration state (Node 2) were enriched in responses to oxidative stress, lignin biosynthetic process, photosynthesis, protein-chromophore linkage, cellular redox homeostasis, and translation. On the other hand, in *H. dentatum*, the dehydrated state (Node 2) was enriched in functional categories related mainly with antioxidant responses such as glutathione metabolic process and ROS detoxification systems, drought response, transcription, translation regulators, photosystems II stabilization, ATP synthesis and proton transport, photoprotection, and ABA non-regulated stress responses. For the rehydrated state (Node 6), transcripts were enriched in functional categories corresponding mainly to ethylene and abscisic acid related signaling, photosynthesis, proton transport, plasmodesmata-mediated intercellular transport, response to stress and to toxic substances.

**Fig. 7:**
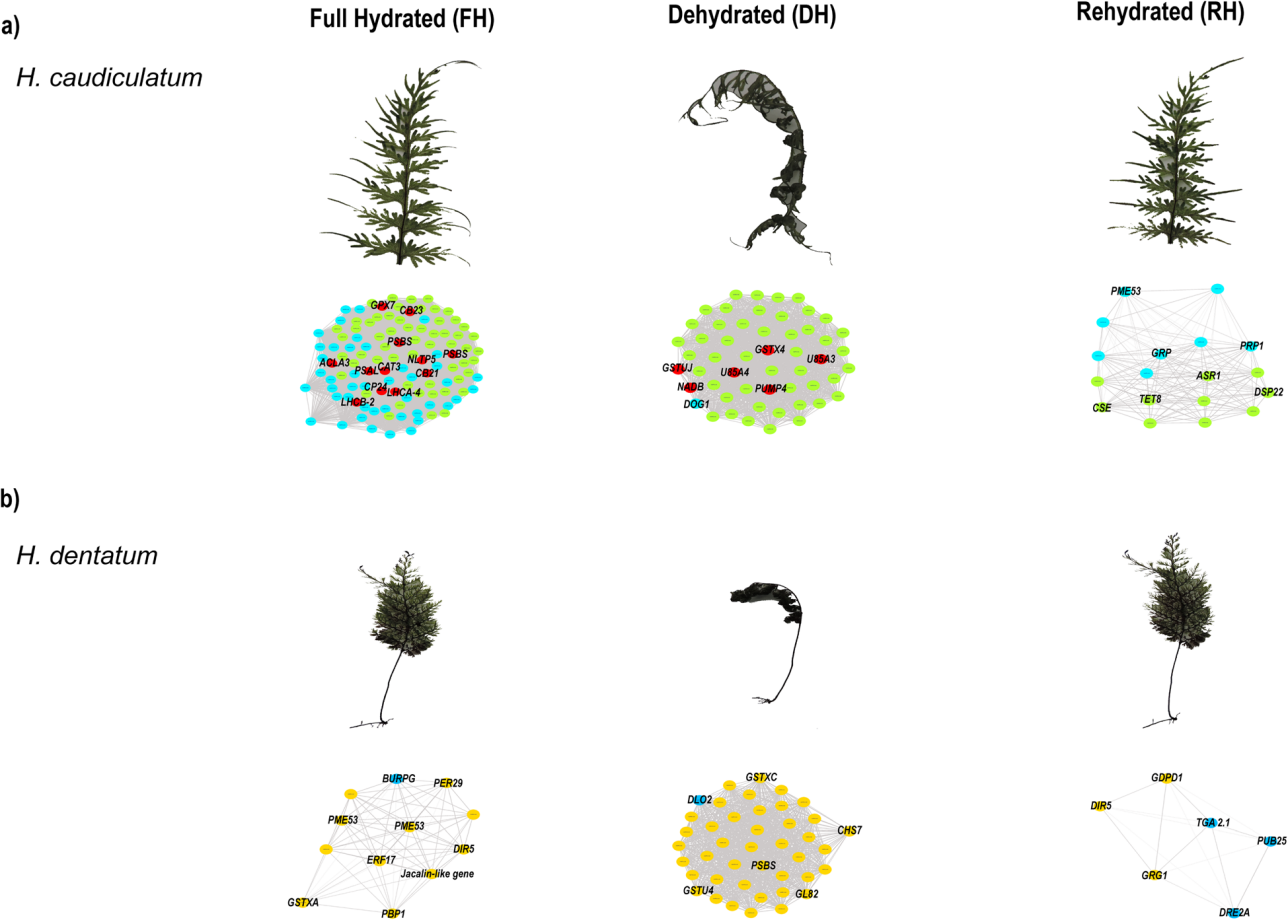
Gene co-expression networks analysis for the full hydrated (FH), dehydrated (DH) and rehydrated (RH) states of (**a**) *H. caudiculatum* and (**b**) *H. dentatum* fronds. The genes used for the network construction were obtained from Nodes 2, 3, and 6 for *H. caudiculatum*, and Nodes 1, 2 and 6 in *H. dentatum* (See Fig. 6b and d). Each network included two modules indicated by the colors of the circles (green and light blue for *H. caudiculatum*, yellow and blue for *H. dentatum*). Hub genes (> 100 connections) are in red. Each of the modules contain transcripts with denser connections representing predicted interactions. The names of those genes showing higher connections within a given hydration state are indicated and their specific functions are discussed on the text

From these SOM nodes (2, 3 and 6 in *H.caudiculatum*, and 1, 2 and 6 in *H. dentatum*), we carefully reviewed the annotated genes with high scale expression to construct weighted gene coexpression networks for each node (Fig. 7; see Materials and Methods for details). Based on the Fast Greedy modularity optimization algorithm for finding community structure, all gene-coexpression networks had two modules. The networks obtained from the full hydrated state of *H. caudiculatum* (Node 6, Fig. 7a), and from the dehydrated state of *H. dentatum* (Node 2, Fig. 7b) showed the highest gene connectivity, i.e., interactions between genes (6216 and 866 connections, respectively). An overview of the resultant gene-coexpression network for each hydration state of *H. caudiculatum* (Fig. 7a) showed that at full hydration, twelve hub genes (> 200 connections) composed a core network connecting both modules, which were involved in: protective system against oxidative stress (*GPX7*, *CAT3*); light harvesting complexes and reaction centers of photosystems I and II (e.g., *CB23*, *CP24*, *LHCA 4*, *LHCB 2*, *PSAA*, *PSBA*); lipid metabolism and transport (*ACLA3*, *NLTP5*). Under desiccation, the resultant network showed six hub genes (> 100 connections), and they were part of one of the modules. Hub genes were involved in: cell wall reinforcement (*WUN1*); glutathione metabolism (e.g., *GSTUJ*, *GSTX4*); mitochondrial uncoupling protein (*PUMP4*); glycosylation (*U85A3*); nitrogen mobilization (*NRT*s). At the rehydration state, the resultant network contained only eighteen genes, all with the same number of connections among them. Two main process were represented by these genes, namely cell wall structure and architecture (e.g., *PME53*, *PRP1*, *CSE*), and stress response and signaling (e.g., *GRP*, *ASR1*, *DSP22*, *TET8*).

Regarding *H. dentatum*, there were no hub genes in none of the resultant gene-coexpression networks (Fig. 7b). At the full hydration state, the network contained twelve coexpressed genes, equally connected among them. They were grouped into different functional categories, such as plant defense and abiotic stress resistance (e.g., *DIR5*, *ERF17*, *BURP16*), chloroplast development (e.g., *PBP1*), and cell wall structure and architecture (e.g., *PME53*). Under desiccation, the resultant network contained a total of forty-two coexpressed genes. Among them, we found genes involved in flavonoid biosynthesis (*CHSY*), structure of photosystems I and II (*PSAA*, *PSBS*), stress response (*GL82*), ubiquitination (*UBIQP*), aldo-keto reductases (*ALKR4*), immune system and Salicylic acid homeostasis (*DLO2*), non-symbiotic hemoglobins (*HBL*) which function in signal transduction pathways of several hormones, Jasmonic acid precursor (*OPR7*). Lastly, at the rehydration state the resultant network contained only 6 coexpressed genes. They were mainly involved in membrane metabolism (e.g., *GDPD*), stress response and signaling (e.g., *DIR5*, *GRG1*, *DRE2A*), and plant immunity (e.g., *PUB25*, *TGA2.1*), and sugar metabolism (*GRG1*).

## Discussion

Previous studies have indicated at different scales, from cellular to ecophysiological approaches, how Hymenophyllaceae species from temperate rain forest responds to desiccation [12, 13, 15–17, 23]. However, the link among desiccation response, habitat preferences and gene expression have never been addressed before in Hymenophyllaceae until our present study. Both de novo assembled transcriptomes (the first for the Hymenophyllaceae family) exhibited good quality parameters, comparable to other non-model species transcriptomes sequenced with the same Illumina platform and using the same criteria for subsequent downstream assembly pipelines [24–26]. Thus, the robustness of our transcriptomes allowed us to explore differential gene expression and to contrast the dynamical patterns of high-and-low abundance of genes during the transition from dehydration to rehydration versus the constitutive gene expression, thereby contributing to a comprehensive view of the response mechanisms involved in a fast dehydration-rehydration process. Finding patterns in massive gene expression data sets consistent with a biological function is always a challenge. Despite the undeniable advantages of exploratory statistical models such hierarchical clustering [27] and *k-*means clustering [28], these methods are subjective due to human bias based on arbitrary statistical significance threshold and does not consider the topology between clusters [29]. The characteristics of the artificial neural networks, considering topology in clusters neighboring [19, 20], provides an excellent tool to depict clustering patterns of gene expression across multiple factors [19, 30, 31]. With the differential gene expression analysis, we found similar patterns of molecular responses between both filmy ferns during the desiccation-rehydration cycle. Then, by combining the Self-Organizing Maps with weighted gene co-expression network analysis, we showed clear patterns of transcript accumulation and found a clear relationship between core genes that are co-expressed in a given hydration state. Thus, we were able to elucidate both shared and species-specific molecular components such as mechanisms of signaling and responses to desiccation [6, 32], disturbance of chloroplast homeostasis [33, 34], and photoinhibition-related stresses [35–37], associated with frond hydration state and the spectrum of microhabitat that *H. caudiculatum* and *H. dentatum* occupy along the vertical distribution over host trees.

At a fully hydrated state, the patterns of transcripts accumulation in both species reflect normal functioning of primary metabolic process such as photosynthesis and respiration. After dehydration, the clustering patterns and abundance of differentially expressed identified transcripts, shared mechanisms associated with the maintenance of redox homeostasis, stabilization and maintenance of the photosynthetic apparatus, and chloroplast operational signaling. In contrast, the species-specific responses were particularly associated to phenolic compounds biosynthetic pathways (e.g., phenylpropanoid metabolism) and photoinhibition related processes. Both general and species-specific responses agree with physiological, ultrastructural, and chemical changes, as with the homoiochlorophyllous strategy of these filmy ferns [11, 13, 15, 23].

### Shared patterns of transcriptional responses during the dehydration-rehydration cycle

It must be noted that because the relatively few transcripts showing differential expression, the desiccation tolerance response of these two filmy ferns would rely mainly on constitutive mechanisms, as it has been suggested by proteomic analyses [16]. Based on our results, both filmy-ferns have high abundance of transcripts involved in translation, photosynthesis and antioxidant capacity regardless the hydration state of the frond. An interesting result from the highly abundant constitutive genes is the expression of the *LATE EMBRYOGENESIS ABUNDANT PROTEIN 14* gene (*LEA14*) which has been reported as an atypical LEA protein localized in the cytoplasm and nucleus, with important roles against abiotic stress, particularly osmotic and drought stress [38, 39].

The patterns of differential gene expression shared by the two species (as shown in Fig. 4) suggest conserved mechanisms of responses regardless their habitat preferences along the vertical gradient of host trees. Thus, during dehydration both species increase the abundance of genes encoding for *Glutathione-S-Transferases* (*GST*), while there is a decrease of *GST* genes following rehydration. GST enzymes scavenge a wide variety of toxic compounds (e.g., hydrophobic, electrophilic and cytotoxic substrates; see [40]). When the fronds of both filmy ferns turn desiccated, they must deal with the cytotoxic effects of reactive oxygen species associated with multiple and simultaneous stress to which they are subjected, such as water deficit and high light stress [41]. Efficient detoxification and antioxidant defense systems are key components for desiccation tolerance [3, 6, 36]., Given that GST enzymes inactivates a wide variety of toxic compounds (e.g., hydrophobic, electrophilic and cytotoxic substrates; see [38]), these two ferns appear to have evolved a mechanism to finely tune the GST responses against oxidative damage during the desiccated state. We found the same dynamic in genes encoding for ferritins which could be a more specific mechanism coupled with the aforementioned for GST. Ferritin is a putative iron storage protein that has been linked with ROS metabolism in plants. Specifically, ferritin synthesis is activated at the transcriptional level by cellular iron and H_2_O_2_ as well as by high light intensity, enhancing ROS detoxifying enzymes [42]. Evidence that links ferritin and ROS metabolism points out that the control of ferritin synthesis would be required for a proper maintenance of the redox status of fronds cells. Moreover, it is known that ferritin domains are part of the structure of desiccation related proteins (DRP, [43]), therefore besides the antioxidant role of ferritin, the two filmy ferns may use this protein for protection of membrane structures, such as photosynthetic structures [44].

Both species also shared patterns of gene expression involved in the control of structure and function of photosystems. During desiccation both species accumulate transcripts encoding for components of reaction centers of Photosystem I and II while there is a decrease in transcripts abundance encoding for light-harvesting antenna and oxygen-evolving complexes. This response differs with what has been reported for the model resurrection plant *Craterostigma pumilum*, in which the inverse pattern occurs [36]. A plausible explanation is that because of the homoichlorophyllous nature of Hymenophyllaceae species, i.e. the retention of their chloroplast when desiccated, this genes dynamics would help during the time-lapse of desiccation-rehydration against the combined effects of severe dehydration and intermittent occurrences of high irradiances inputs by sunflecks to deal with reactive oxygen species, oxidative damage [45], and the need to minimize the potential for light capture and the oxidation of water in order to decrease redox reactions but to maintain a stock for reaction centers.

The coordination of the complex mechanisms such as those mentioned above require intertwined signaling pathways for systemic responses against the internal and external constraints associated with the resurrection strategy. The increases of transcript abundance of *AAA-ATPase* genes which are known to be involved in ROS signaling and the presence of components of signal transduction such as Auxin and calcium signaling, oxylipin metabolism, and ethylene and ABA responsive transcription factors points to the chloroplast as the signaling orchestrator [34]. Because the fronds of these ferns ranges from one to a few cells layers, and given that plastid-to-nucleus signaling is essential for the coordination and adjustment of cellular responses to external and internal cues [33, 46, 47], environmental perturbations imposing restrictions for the chloroplast homeostasis would have a great impact in whole frond form and function. Fluctuations in frond water status will initiate an operational signaling to change the expression of thousands of genes to adjust chloroplast and the whole cell functioning [34]. According to this, both species showed an increase of transcript abundance that encode for AP2/ERF transcription factors during desiccation (e.g., *DREB*, *ERF*, *RAP*), which are known to be involved in responses to environmental stimuli, especially for redox, osmotic and drought stress [33, 34]. Moreover, a coordination of chloroplastic and nuclear gene expression would be mediated by the redox status [48], activating a protein kinase phosphorylation cascades and AP2/ERF transcription factors. Our results are consistent with the above-mentioned mechanism, being a key mechanism contributing to the resurrection strategy used by the two studied filmy ferns.

### Species-specific patterns of transcriptional responses during dehydration and rehydration

One of the most ambitious goals of our study was to associate the different niche preference of these two filmy fern species with species-specific transcriptional responses to have insights into particular physiological conditions that may prevail under different frond hydration states. In general, based on the DE analysis and the pairwise comparison among hydration states (Table 1), the specific responses in *H. caudiculatum* underpins enhanced osmotic responses and phenylpropanoid related pathways, whereas in *H. dentatum* the specific responses point to enhanced protection against oxidative damage and high light stress. By the other hand, the gene co-expression networks analysis provides a detailed interaction of central genes conferring each species-specific response. Examining the responses of *H. caudiculatum* during the dehydration-rehydration cycle, we observed an increase in the abundance of the *DOG1* gene when full hydrated and rehydrated. This gene is involved in controlling the timing of seed dormancy in response to environmental signals such as temperature by altering gibberellin metabolism [49]. Given that during embryo development and maturation the acquisition of desiccation tolerance is a key process for the viability of the majority of seeds, it is logical that resurrection plants use part of the molecular components involved in the control of seed dormancy. However, the changes in the abundance of *DOG1* gene in *H. caudiculatum* suggest an induced mechanism linked to ABA and GA balance by a common intermediary such as DELLAs proteins [50]. Based on the gene-coexpression network results, we observed for the dehydration state that core genes are related to minimize ROS formation and oxidative stress through NAD metabolism and mitochondrial uncoupling proteins [51, 52], while for rehydration all co-expressed genes are involved mainly in cell wall and protein stability [53], membrane stability and signaling, and the reestablishment of glucose metabolism (Fig. 7a).

**Table 1:** Differential expression of transcripts of interest (FDR < 0.05) during experimental desiccation-rehydration cycle in *H. caudiculatum* and *H. dentatum* fronds. The table shows the pairwise comparison of the increase/decrease abundance of transcripts between the transition of fronds from one to another hydration state, namely full hydrated (FH), dehydrated (DH, and rehydrated (RH). Gene identities were obtained with GO-enrichment by Panther Classification System V11.0. The level of regulation of each gene was estimated by value of the logarithm of fold change (logFC, on brackets) above and below 1.0 and − 1.0, for up and down regulation, respectively

Species	Abundance	Pairwise comparison between hydration states		
		FH x DH	DH x RH	FH x RH
		Annotated Genes (logFC)	Annotated Genes (logFC)	Annotated Genes (logFC)
*H. CAUDICULATUM*	Increase	*AKR1(2.2); AKR2(1.8); ALKR3(1.9); ALKR4(1.8); ALL12(4.8); AMT12(3.4); ATPA(3.0); ATPB(5.0); ATPF(3.0);ATPH(3.0); ATPI(3.0); CEMA(5.0); CHLB(3.0); CLPP(5.0); BAM1(1.6); BGL25(5.0); BURPG(1.8); CADH(2.5); CB2(9.6); COMT1(1.9); COR2(3.1); CYB6(5.0); CYF(5.0); DOG1(2.1); ERF17(1.9); ERF55(2.2); ERF80(2.7); FAD4(1.9); FRI3(3.0); GDPD1(2.8); GSTF3(2.3); GSTFA(2.3); GSTL2(2.5); GSTUH(3.0); GSTUJ(3.9); GSTUK(2.6); GSTUS(1.9); GSTX2(2.1); GSTX4(4.4); H2A7(1.8); HACL(1.9); HBL(2.7); HSP7C(2.0); HST(2.4); IAA17(2.1); IN21B(3.2); IQD1(2.0); MASY(3.4); MPCP3(3.0); NAC68(3.5); NADB(2.6); NDHJ(2.3); NDHK(2.3); NIR(2,8); NRT24(7.0); NRT31(3.2); NU3C(2.3); OPR11(2.3); OPR5(1.8); PCKA(3.1); PETD(5.0); PLA5(2.2); PMA4(1.8); PSAA(1.7); PSBA(3.0); PSBB(5.0); PSBE(5.0); PSBH(5.0); PSBK(3.0); PBSN(5.0); PUMP4(4.5); QORL(2.0); RA213(2.1); RBL(5.0); RUBREDOXIN(2.3); SAU72(1.8); SRC2(3.6); U85A1(2.2); U85A2(2.4); U85A3(3.7); U85A4(2.9); U85A7(1.9);UB6A1(2.3); WUN1(3.0); YAA4(2.0); YCF2(3.0); YCF3(2.3); YCF4(5.0)*	*ASR2(4.2); AXS1(2.4); CB23(3.9); CFI3(2.5); CHSY(4.0); EXTENSIN1(10.7); GDL10(5.6); GRP(9.3); MYR1(2.9); PALY(4.0); PGL2(3.6); PME53(5.2); PPO(2.9); PRP1(11.1); SHT(2.5); U85A2(2.8)*	*AAP4(2.2); AB2C(1.9); ACCD(5.5); AKR1(2.4); ALL12(3.7); ARP(2.1); ASR1(2.7); ATPA(3.5); ATPB(5.8); ATPE(5.8); ATPF(3.5); ATPH(3.5); ATPI(3.5); BAM1(2.2); BGL25(4.7); BURPG(1.8); CB2(9.7); CEMA(5.5); CHLB(3.5); CLPP(5.5); COX2(2.5); COX3(2.6); CSE(2.9); CYB(2.2); CYB6(5.5); CYF(5.5); DIR1(2.1); DOG1(2.5); DS22(1.8); ERF17(2.2); ERF80(2.7); FAD4(2.4); FRI3(3.1); GRP(3.5); GSTFA(2.6); GSTL2(3.0); GSTUH(2.1); GSTUJ(4.0); GSTX4(3.6); IFRH(2.2); IN21B(2.6); MASY(3.0); MECR(3.9); MFT(2.1); MI25(2.6); MPCP3(2.4); NDHJ(2.4); NDHK(2.4); NRT24(3.1); NU3C(2.4); NU3M(2.1); OPR11(2.4); OPR5(2.2); PCKA(3.5); PETD(); PRP1(1.9); PSAA(2.1); PSAB(2.0); PSBA(3.5); PSBB(5.8); PSBE(5.8); PSBH(5.8); PSBK(3.5); PSBN(5.8); QORL(2.0), RBL(5.8); RCI2A(1.9); RK14(5.8); RR11(5.8); RR14(2.1); RR19(5.8); RR3(5.8); RR4(2.4); RUBREDOXIN(2.8); SRC2(4.5); TCMO(5.3); TET8(1.9); YCF2(3.5); YCF3(2.4); YCF4(5.8)*
	Decrease	*AATPD(-2.6); ALF1(-2.1); ARF1(-3.2); ASPG2(-1.9); ASR2(-5.7); AXS1(-3.1); BCA2(-1.9); CA4(-2.0); CATA3(-2.7); CB12(-2.6); CB121(-1.9); CB13(-1.8); CB2(-3.1); CB21(-3.7); CB23(-5.7); CB4A(-2.7); CB4B(-2.6); CB5(-2.2); CCH(-2.3); CFI(-1.7); CFI3(-3.2); CHSY(-5.2); CRD1(-1.9); CRSP(-6.9); CUT1A(-2.2); D5FAD(-2.2); DFRA(-2.6); DHAR2(-11.7); DRPE(-2.2); EXTENSIN1(-11.0); FTRC(-1.8); GDL10(-7.2); GDL61(-1.8); GPX7(-2.1); GRP(); GRS17(-3.7); GSA(-2.1); GSTX4(-1.7); LEA4(-X.X); LTPG2(-2.0); MYR1(-4.1); NLTP5(-2.4); NLTP6(-2.3); P2SAF(-1.7); PADC(-6.7); PALY(-4.7); PB27A(-2.1); PER29(-2.5); PGL2(-4.1); PPO(-3.4); PRE2E2(-2.1); PRX2B(-3.6); PSAH(-1.7); PSAL(-1.8); PSBO(-6.7); PSBP(-1.9); PSBQ2(-2.1); PSBY(-1.8); PSBX(-3.5); SSG1(-2.2); TLPH(-4.4); U85A1(-2.6);U85A2(-5.0)*	*ADT1(-3.1); ALKR4(-2.3); AMT12(-2.8); COMT1(-2.1); HBL(-2.8); HST(-1.5); NAC68(-3,5); NIR(-3.1); NRT24(-3.9); NRT31(-2.5); PLA5(-2.1); PUMP4(-2.7); TCMO(-2.6); U85A2(-3.0); U85A3(-2.5); U85A4(-2.3)*	*AATPD(-3.3); CATA3(-1.9); CB12(-2.5); CB2(-2.2); CB21(-2.5); CB23(-4.8); CB4A(-2.2); CB5(-2.1); CP74(-2.0); CRSP(-5.8); CUT1A(-2.2); DHAR2(-16.1); DRPE(-2.6); GALDH(-1.9); GRS17(-3.1); GSA(-2.0); HSP12(-2.0); OSL3(-3.6); PADC(-2.4); PAP14(-2.0); POR(-3.6); PR2E2(-2.0); PRX2B(-3.0); PSBO(-4.8); PSBQ2(-1.9); SDC3(-1.8); U85A2(-2.3); U85A3(-2.1)*
*H. DENTATUM*	Increase	*AAP4(2.4); ALDH(2.2); ALKR3(1.9); ALL12(2.2); ASR1(2.1); ATPA(2.9); ATPAM(2.3); ATPH(3.4); ATPI(3.4); CATA2(2.1); CHLB(2.9); DRE2A(2.2); EF1A(3.2); ELI6(2.5); ERF17(3.1); FRI3(2.4); GSTU4(2.7); GSTUH(2.1); GSTUK(1.9); GSTXA(2.1); HBL2(2.0); HIP26(2.2); LTP2(2.5); MASY(1.8); OMT1(2.7); OPR7(2.7); PCKA(3.1); PIR3(2.0); PSAA(2.5); PSAB(2.3); PSBA(2.9); PSBK(2.9); RA213(2.8); U85A1(2.8); UBI1P(2.4); UBIQP(2.9); ZB14(2.9)*	*ASR1(3.4); ATCA7(2.1); BURPG(2.6); CYSEP(3.2); EF1A(5.9); ERF18(2.3); FT(2.2); GDPD1(4.1); HIP26(2.6); ISCAP(2.6); LACP(6.9); PBP1(2.6); PER29(2.0); RCI2A(2.0); SRP(2.8)*	*CYSTM(9.1); DRE2A(3.1); EF1A(2.3); ELI6(3.0); ERF17(2.9); GDPD1(2.2); HIP26(2.3); RA213(2.1); TGA21(2.2); UBIQP(4.0)*
	Decrease	*ASR1(-3.0); ASR2(-2.0); ATCA7(-2.5); BURPG(3.8)-; CB23(-1.9); CYSEP(-3.7); DHAR2(-1.8); DIR5(-2.0); EF1A(-6.3); ERF18(-2.1); GDPD1(-1.9); HIP26(-1.9); ISCAP(-2.8); LACP(-4.5); LIN1(-7.2); NLTP(-2.0); PBP1(-2.0); PER29(-3.1); PME53(-2.6); PSBO(-1.9); RCI2A(-1.9); REHY(-2.0); SRP(-2.0); YLS9(-2.0)*	*ALKR3(-2.8); ALL12(-3.0); ASR1(-2.0); ATPA(-1.8); ATPAM(-1.8); ATPH(-3.0); ATPI(-3.0); CALM(-2.0); CHLB(-2.9); CHS7(-4.6); CLH1(-2.8); DBR2(-2.2); FRI3(-2.2); GL52(-3.1), GL82(-2.7); GSTU4(-3.8); GSTUH(-3.3); GSTUK(-1.9); GSTXA(-3.2), HBL2(-2.0); IN21B(-1.9); LOX2(-1.9); OPR5(-1.9); OPR7(-2.4); PCKA(-2.5); PIP28(-2.3); POLX(-2.0); PSAA(-2.6); PSAB(-2.3); PSBA(-2.9); PSBK(-2.9); PSBS(-2.1); U85A1(-2.2); ZB14(-2.9)*	*CHS7(-4.2); CLH1(-2.6); DIR(-2.2); GL52(-2.4); GSTFA(-2.0); GSTXC(-3.5); PME53(-2.5)*

We observed an increase in the abundance of the *ELI6* gene which encodes an early light inducible protein (Table 1) in *H. dentatum* when full hydrated and rehydrated. The same patter was observed for the dehydration response element transcription factor *DRE2A*. ELIPs proteins are induced by light, and act as sinks for excitation energy under high light. The increase in abundance *ELI6* and *DRE2A* only in *H. dentatum* (Fig. 7b) would be a response associated with its distribution toward more illuminated and low humidity micro-sites along the vertical direction of host trees. Other specific responses are linked with the activation of defense responses driven by Ethylene and Salicylic Acid, and the scavenging of specific ROS such as hydrogen peroxide.

## Conclusion

The present work provides a deeper understanding of the mechanisms underlying the desiccation tolerance responses of *H. caudiculatum* and *H. dentatum*. Here we have deciphered the transcriptional dynamics, gene co-expression networks and key genes involved in the signaling and response mechanisms during the process of dehydration and rehydration for both species. In addition, the identification of species-specific mechanisms contributes to explain their ecophysiological traits and microhabitat preferences they show in their natural environment. Thus, while the lower-canopy species *H. caudiculatum* seems to enhance osmotic responses and phenylpropanoid related pathways, the upper-canopy species *H. dentatum* enhanced its defense system responses and protection against high light stress.

Finally, much more work is needed to understand the roles and contribution of specific molecules (e.g., RNA-binding proteins) and posttranscriptional regulations that may have central roles in the desiccation tolerance strategy of these species when subjected to rapid loss-and-gain of water as occurs in the natural environment. Also, studies under an evolutionary developmental biology approach would help to decipher and understand the genetic basis of physiological, developmental and morphological variation associated with the microclimatic conditions in which these resurrection filmy ferns have evolved.

## Methods

### Plant material and growth conditions

Individuals of *Hymenophyllum caudiculatum* (Mart.) var. productum (K*.* Presl.) and *Hymenophyllum dentatum* (Cav.) (Fig. 1) were collected from Katalapi Park (41°31′07.5″ S, 72°45′2.2″ W) Cordillera de Quillaipe, Los Lagos Region, Chile. The collecting site correspond to a coastal evergreen temperate rainforest. Details on microclimatic conditions of the site have been previously published [11]. Small pieces of bark or fallen trees covered with epiphytic filmy ferns were collected in late Spring (Nov-Mid Dec, Southern hemisphere) and transported to a shaded experimental nursery garden with automated irrigation at the Universidad de La Frontera. Further description of nursery conditions and plant set up can be found in [17]. Briefly, plants were acclimated to nursery garden conditions under shade (25–30 μmol photons m⁠ − 2 s⁠ − 1), nebulized by an automated irrigation system (6 daily waterings, 2 min each) for 25 days prior to experimental procedures.

It is worth to note that the deposit of specimens to an herbarium was not necessary, since they already exist in the collection of the Herbarium of the Universidad de Concepción (http://www2.udec.cl/~herbconc/index.htm, Herbario CONC, Departamento de Botánica, Universidad de Concepción, Barrio Universitaro s/n, Casilla 160-C, Concepción, Chile.)

### Experimental design and sample collection

The experimental design was a desiccation-rehydration experiment under nursery conditions during late spring (Nov-mid Dec, southern hemisphere) in which fronds of *H. dentatum* and *H. caudiculatum* were studied at three different hydration states; full hydrated (FH), dehydrated (DH), and rehydrated (RH) (detailed in Fig. 2a). At each hydration state, we used attached fronds for monitoring the changes in maximal quantum efficiency (Fv/Fm), and two set of detached fronds; one to determine their relative water content (RWC), and the other for RNA isolation. To reach a fully hydrated state, ferns were subjected to irrigation pulses of three minutes at intervals of twenty minutes each, during four hours; a single frond from each plant (3 plants per species, 6 plants total) were taken at this hydration state (FH) to obtain its relative water content (RWC). Then, ferns went through a desiccation-rehydration process by adjusting the irrigation settings and monitoring the changes in RWC and Fv/Fm of the fronds during the next seven days. AT the first day without irrigation, the first DH sampling (fronds from three different individuals per species) was taken when fronds reached about 60% of RWC. A second DH sample was obtained when plants reached a value equal or below the critical relative water content reported for these species [11], achieved at day seven (Fig. 2a). Both DH samples were pooled in order to have coverage for early and late desiccation-regulated transcripts. Finally, the rehydration was carried out resuming irrigation until at least a 90% of RWC was recovered. During rehydration (RH) two samplings were taken, one was obtained when plants recovered 60–70% of RWC, and the second sampling was taken at ca. 90% of RWC. Both samplings were pooled as RH in order to have coverage for early and late rehydration-regulated transcripts. Samples were stored at − 80 °C until processed for RNA isolation for comparisons of the transcriptional responses associated to each hydration state, either within or between species.

### Fluorescence measurements

Maximal quantum efficiency (Fv/Fm) was calculated from chlorophyll *a* fluorescence signals obtained from attached fronds using a modulated fluorometer (FMS2, Hansatech, UK) as previously described in [17]. Briefly, attached fronds were carefully cover and dark acclimated for 30 min using FMS2 leaf clips. After dark acclimation the modulated light was turned on to obtain F0, then a saturating pulse (800 mS at ~ 3000 μmol m^− 2^ s^− 1^) was applied to obtain the maximum fluorescence (Fm). Variable fluorescence (Fv) and Fv/Fm ratio were calculated according to [54] at ambient temperature (17 C° approx.). Data was analyzed after check normality assumptions under an ANOVA test with *P* value ≤0.05. When data did not meet the normality assumptions, we used the non-parametric test of Kruskal-Wallis. Additionally, fluorescence images of Fv/Fm were obtained using detached fronds under each hydration condition FH, DH and RH using a Maxi-Imaging PAM (Walz, Effeltrich, Germany) to observe the rate of recovery of the quantum yield of fluorescence (YII) at the whole frond level of both species (Fig. 2b).

### RNA-seq and *De novo* Transcriptome analysis

Total RNA was isolated using UltraClean™ Plant RNA Isolation Kit (Mo Bio, *Carlsbad*, *CA*, *USA*) and purified with Total RNA I kit (Omega Bio-Tek, Norcross, GA, USA) according to manufacturer’s instructions. The yield and quality of the RNA isolation samples was determined by an Agilent 2100 Bioanalyzer. The RNA was precipitated with two volumes of acetate:ethanol solution (1:10 v/v) and sent for sequencing to Macrogen Inc., Korea. A total of six samples were sequenced in a single lane of an Illumina HiSeq 2000 platform (Illumina Inc. San Diego, CA, USA) obtaining 100 bp paired end reads. After quality filtering with the NGSQC Toolkit v2.3 ([55], http://www.nipgr.res.in/ngsqctoolkit.html) and Q-score composition (Additional file 3: Figure S1), we conducted a *de novo* assembly with the Trinity software package v2.1.1 [56, 57]. Transcriptome assembly was performed at Troquil Linux cluster at Centro de Modelación y Computación Científica (CMCC, Universidad de La Frontera) using 12 processors Intel Xeon E5–4640 and 192 GB of shared memory.

Reads from each sample were mapped to their corresponding final transcriptome using default RSEM parameters (RSEM = RNA-Seq by Expectation Maximization) written in the align_and_estimate_abundance.pl script. The resulting RSEM-estimated gene abundances for each fern were merged in a matrix and analyzed with run_DE_analysis.pl script from Trinity, which involves the Bioconductor package edgeR in R statistical environment [58, 59]. Transcripts with very low estimated counts (2 for combined groups), were not considered for edgeR pair-wise comparison of hydration states. To judge significance of gene expression, we used a False Discovery Rate value (FDR) lower than 0.05 and a minimum fold change (FC) of 2 as thresholds (Additional file 2: Dataset S1 and S2). To quantify transcript abundance, normalized RSEM-estimated counts were used for clustering assembled contigs based on expression patterns [29]. Finally, the resulting transcripts of each transcriptome were aligned into the SwissProt database using BLAST+ with an e-value filter of 1-e^− 10^ as threshold [60]. Functional annotation, classification, and over-or under-represented groups of genes were performed using PANTHER ([61], www.pantherdb.org).

### Self-organizing maps (SOM) analysis

Normalized RSEM-estimated counts of both *H. caudiculatum* and *H dentatum* that met the expression values determined from the model described above were used for the SOM clustering method [29]. Specifically, only genes that vary significantly in expression across fronds hydration state of both filmy fern species were analyzed. To focus only on gene expression profile, and at the same time avoid biases with the differences in the magnitude of gene expression, expression values were mean centered, selected from the upper 50% quartile of coefficient of variation, and variance scaled using the scale function (R base package [58];) separately for *H. caudiculatum* and *H. dentatum*. Scaled expression values were used to cluster genes in both species across fronds hydration states into a multidimensional 2 × 3 hexagonal SOM using the Kohonen package on R [19]. One hundred training iterations were used during clustering with a decrease in the alpha learning rate from ca. 0.0018 to 0.0010 (Additional file 3: Figure S2). After the iteration process, the final assignment of genes to the winning units shaped the clusters of genes (termed nodes) associated to the hydration states. SOM outcome was visualized into pie charts for codebook vectors to obtain the counts number and mean distance of the genes assigned to each node ([19], Additional file 3: Figure S3). The box plot option from the ggplot2 package on R was used to visualize the gene accumulation patterns associated to the hydration states of the fronds in each Node. Finally, the genes of each Node were analyzed for GO enrichments terms at a 0.05 false discovery rate cutoff (Additional file 4: Dataset S6 and S7).

### Gene co-expression network analysis

In order to go further the gene co-expression patterns obtained from the SOM analysis, a further Gene Regulatory Network-based (GRN) approach was used to study the interactions between gene expression and fronds hydration state of each fern species. From the SOM clustering method, a subset of 67 and 183 annotated genes from *H. dentatum* and *H. caudiculatum* were selected, respectively. For the selection of genes, those transcripts from SOM nodes with the highest scaled accumulation patterns for a given hydration state were chosen. Then, only those genes with GO annotation were selected. (Additional file 2: Dataset S4 and S5). These genes were used to construct a weighted gene co-expression network according to [31]. Briefly, features of two R packages, namely, Weighted Gene Coexpression Analysis (WGCNA [18];) and igraph [21], were combined to visualize the genes interaction. To calculate the adjacency matrix with the WGCNA package, a soft threshold (β) value of 9 was used to achieve the scale free topology criteria. Then, the network connectivity and modularity were visualized by using the algorithms of the graph generator and community structure functions of the igraph package, whilst custom graph functions were used for network visualization (available at https://github.com/eostria/Gavel).

### Sequence submission

The quality-filtered, barcode-sorted, and trimmed short read data set used for transcriptome assembly and gene expression analysis, was deposited in the NCBI Sequence Read Archive (SRA) under accession SRR5195043, SRR5272488, SRR5272490, SRR6452149, SRR6452987, and SRR6453153. Additionally, the gene expression profiles of the assembled transcriptomes were deposited in the NCBI Gene Expression Omnibus database (GEO). For confidential access to the public GEO records use the following links: https://www.ncbi.nlm.nih.gov/geo/query/acc.cgi?acc=GSE140234 and https://www.ncbi.nlm.nih.gov/geo/query/acc.cgi?acc=GSE140238.

## Supplementary information

**Additional file 1:** **Table S1.** Statistics for RNA-seq libraries sequencing of *H. caudiculatum* (Hca) and *H. dentatum* (Hdent) for each of their hydrated states. Statistics shows total number of reads (*No. of reads)*, high quality reads that passed quality control (*No. of HQ reads*), reads that were mapped to transcriptomes (*No. of mapped reads)* and their percentage of mapping (*Mapping rate*) **Table S2.** Assembly statistics for *H. caudiculatum* and *H. dentatum* transcriptomes before (*raw*) and after refinement (*filtered*). The total number of transcripts (*No. transcripts*), number of trinity components (*Trinity components*), putative transcriptome in mega base pairs (*Transcriptome Size*), basic statistics on transcript lengths (*Average, median, minimum and maximum*) and assembly quality as N50 (*N50*) **Table S3.** Blast results for *H. caudiculatum* and *H. dentatum* against SwissProt database. Table shows the counts distribution per sequence size (*Size range*) of blast hit (*Blast Hit*) and missing hits (*Unknow*) and the cumulative percentages (*Cums %*)

**Additional file 2: Dataset S1.** Matrix of the DE genes among pairwise comparissons between frond's hydration states of *H. caudiculatum*. Columns indicates de sequnce ID (id), fold change (log FC), counts per million (log CPM), the significance (PValue), the false discovery rate value (FDR), and the normalized counts for each hydration state (HcaFH.matrix, HcaD.matrix, and HcaRH.matrix), **Dataset S2.** Matrix of the DE genes among pairwise comparissons between frond's hydration states of *H. dentatum*. Columns indicates de sequnce ID (id), fold change (log FC), counts per million (log CPM), the significance (PValue), the false discovery rate value (FDR), and the normalized counts for each hydration state (HdFH, HdD, and HdRH), **Dataset S3.** Matrix showing those highly abundant genes without significant changes between frond's hydration states of *H. caudiculatum* and *H. dentatum*. Columns shows the gene id, transcript id, the counts abundance for each hydration state, the UniProt ID, the percentage of similitud of the Blast query, and the Protein Name, **Dataset S4.** Matrix of the Self-Organizing Maps output for the Nodes 2, 3 and 6 for *H. caudiculatum*, **Dataset S5.** Matrix of the Self-Organizing Maps output for the Nodes 1, 2 and 4 to 6 for *H. dentatum*.

**Additional file 3 **: **Figure S1.** Quality scores and accuracy of Illumina Hiseq 100-bp paired end reads for *H. caudiculatum* and *H. dentatum* after quality filters. Bad quality reads that showed values 20 on the histogram were discarded. All reads above the value of 30 indicates 99.9% of accuracy, regarding to bases correctly read by the sequencer, **Figure S2.** Training progress of the average distances of genes of *H. caudiculatum* and *H. dentatum* using Self-Organizing Maps showing the effect of neighborhood shrinking to include the winning unit, i.e., when the vectors in the dataset reach the closest similarity. **Figure S3.** Codebbook vectors for H. caudiculatum and *H. dentatum* showing the clusters of differentially expresed genes with maximum neighbouring after training process. The codebook vectors represent the expression profile of genes associated to a given state after the constructionof the map

**Additional file 4: Dataset S6.** Compiled GO enrichment data for *H. caudiculatum*, **Dataset S7.** Compiled GO enrichment data for H. dentatum.

## Data Availability

The transcriptomes of the ferns species used in this study are available in the NCBI Sequence Read Archive (SRA) under accession SRR5195043, SRR5272488, SRR5272490, SRR6452149, SRR6452987, and SRR6453153. The gene expression datasets are available at https://www.ncbi.nlm.nih.gov/geo/query/acc.cgi?acc=GSE140234 and https://www.ncbi.nlm.nih.gov/geo/query/acc.cgi?acc=GSE140238. The custom script used for the analysis and construction of the gene co-expression networks is available at https://github.com/eostria/Gavel. All data generated and analyzed in this study are available and included in this manuscript, as well as its supplementary information files.

## Abbreviations

BP: Biological Process
CC: Cellular Component
DE: Differential expression
DH: Dehydrated
FDR: False Discovery Rate
FH: Full Hydrated
FPKM: Fragments per kilobase of exon model per million reads
Fv/Fm: Maximum quantum efficiency
GO: Gene Ontology
MF: Molecular Function
PFD: Photon flux density
RH: Rehydrated
ROS: Reactive Oxygen Species
RSEM: RNA-Seq by Expectation Maximization
RWC: Relative Water Content
SOM: Self-Organizing Maps
WGCNA: Weighted Gene Co-expression Network Analysis
