# Supplementary material for: A comparative gene co-expression analysis using self-organizing maps on two congener filmy ferns identifies specific desiccation tolerance mechanisms associated to their microhabitat preference
Source: BMC Plant Biol. 2020 Feb 4;20:56. doi: 10.1186/s12870-019-2182-3 (PMC7001327; doi:10.1186/s12870-019-2182-3)
Supplement: Supplementary file 1 — Additional file 1: Table S1. Statistics for RNA-seq libraries sequencing of H. caudiculatum (Hca) and H. dentatum (Hdent) for each of their hydrated states. Statistics shows total number of reads (No. of reads), high quality reads that passed quality control (No. of HQ reads), reads that were mapped to transcriptomes (No. of mapped reads) and their percentage of mapping (Mapping rate) Table S2. Assembly statistics for H. caudiculatum and H. dentatum transcriptomes before (raw) and after refinement (filtered). The total number of transcripts (No. transcripts), number of trinity components (Trinity components), putative transcriptome in mega base pairs (Transcriptome Size), basic statistics on transcript lengths (Average, median, minimum and maximum) and assembly quality as N50 (N50) Table S3. Blast results for H. caudiculatum and H. dentatum against SwissProt database. Table shows the counts distribution per sequence size (Size range) of blast hit (Blast Hit) and missing hits (Unknow) and the cumulative percentages (Cums %) [file 12870_2019_2182_MOESM1_ESM.zip › Additional information 1_TableS3.docx]

Supplementary Table 3.

Blast results for *H. caudiculatum* and *H. dentatum* against SwissProt database. Table shows the counts distribution per sequence size (*Size range*) of blast hit (*Blast Hit*) and missing hits (*Unknow*) and the cumulative percentages (*Cums %*).

|  | *Htrymenophyllum caudiculatum* | | | | *Hymenophyllum dentatum* | | | |
| --- | --- | --- | --- | --- | --- | --- | --- | --- |
| Size range | Blast Hit | Cums % | Unknow | Cums % | Blast Hit | Cums %. | Unknow | Cums % |
| 200:399 | 1560 | 8.26 | 4927 | 31.08 | 5906 | 17.35 | 14583 | 41.02 |
| 400:599 | 1381 | 15.58 | 2559 | 47.23 | 5239 | 32.74 | 7171 | 61.19 |
| 600:799 | 1222 | 22.06 | 1618 | 57.43 | 3792 | 43.87 | 4089 | 72.69 |
| 800:999 | 1198 | 28.41 | 1218 | 65.12 | 2833 | 52.20 | 2502 | 79.73 |
| 1000:1199 | 1227 | 34.91 | 1028 | 71.60 | 2265 | 58.84 | 1871 | 84.99 |
| 1200:1399 | 1314 | 41.87 | 826 | 76.82 | 2045 | 54.86 | 1360 | 88.81 |
| 1400.1599 | 1339 | 48.97 | 741 | 81.49 | 1907 | 70.46 | 1034 | 91.72 |
| 1600:1799 | 1334 | 56.04 | 586 | 85.19 | 1679 | 75.39 | 722 | 93.75 |
| 1800:1999 | 1287 | 62.86 | 487 | 88.26 | 1574 | 80.01 | 494 | 95.14 |
| 2000:2199 | 1155 | 68.98 | 384 | 90.68 | 1323 | 83.90 | 388 | 96.23 |
| 2200:2399 | 942 | 73.97 | 302 | 92.59 | 990 | 86.81 | 303 | 97.09 |
| 2400:2599 | 849 | 78.47 | 232 | 94.05 | 822 | 89.22 | 238 | 97.76 |
| 2600:2799 | 678 | 82.06 | 173 | 95.14 | 733 | 91.37 | 146 | 98.17 |
| 2800:2999 | 551 | 84.98 | 144 | 96.05 | 572 | 93.05 | 139 | 98.56 |
| 3000:3199 | 484 | 87.54 | 108 | 96.73 | 426 | 94.30 | 84 | 98.79 |
| 3200:3399 | 373 | 89.52 | 108 | 97.41 | 356 | 95.35 | 98 | 99.07 |
| 3400:3599 | 324 | 92.23 | 66 | 97.83 | 293 | 96.21 | 56 | 99.22 |
| 3600:3799 | 289 | 92.77 | 67 | 98.25 | 263 | 96.98 | 49 | 99.36 |
| 3800:3999 | 252 | 94.10 | 54 | 98.59 | 196 | 97.56 | 43 | 99.49 |
| 4000+ | 1113 | 100.00 | 223 | 100.00 | 831 | 100.00 | 183 | 100.00 |
