# Supplementary figures and images for: A comparative gene co-expression analysis using self-organizing maps on two congener filmy ferns identifies specific desiccation tolerance mechanisms associated to their microhabitat preference
Source: BMC Plant Biol. 2020 Feb 4;20:56. doi: 10.1186/s12870-019-2182-3 (PMC7001327; doi:10.1186/s12870-019-2182-3)

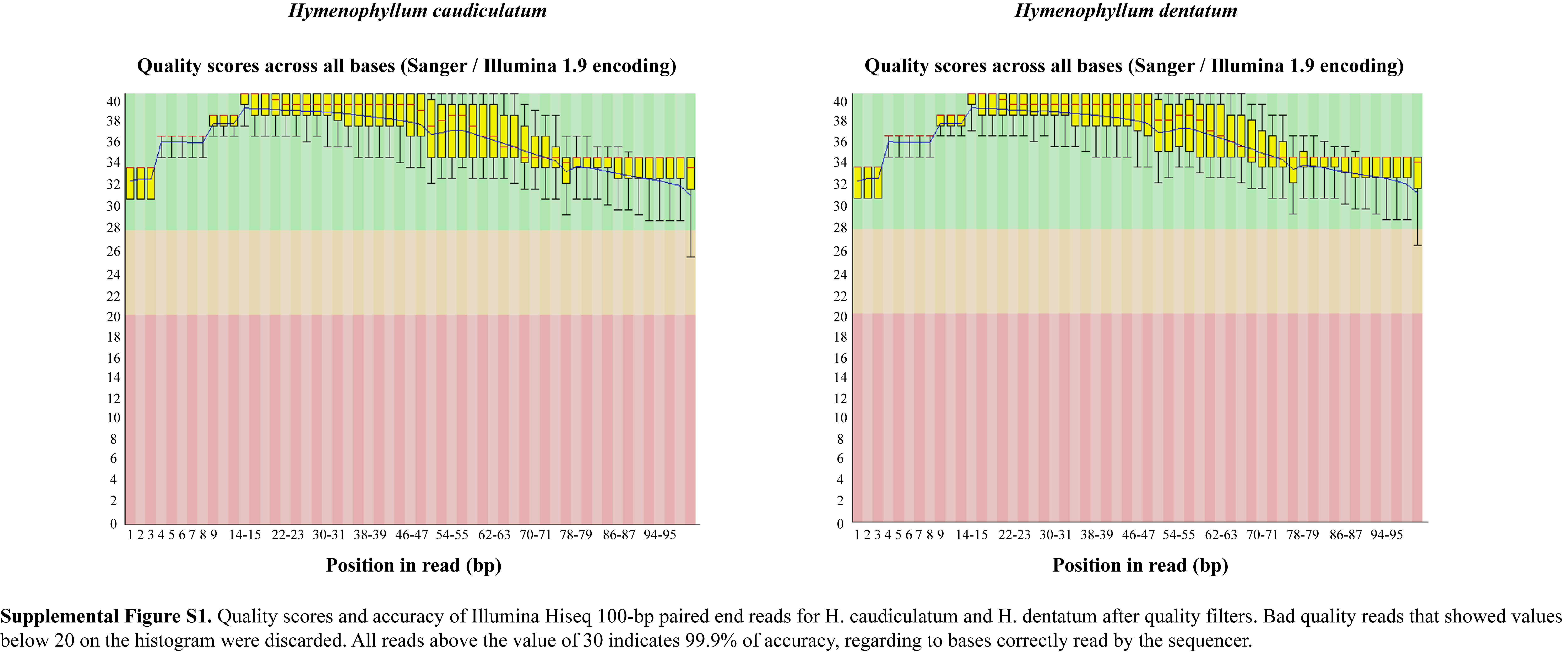

Supplement: Supplementary file 3 — Additional file 3 : Figure S1. Quality scores and accuracy of Illumina Hiseq 100-bp paired end reads for H. caudiculatum and H. dentatum after quality filters. Bad quality reads that showed values 20 on the histogram were discarded. All reads above the value of 30 indicates 99.9% of accuracy, regarding to bases correctly read by the sequencer, Figure S2. Training progress of the average distances of genes of H. caudiculatum and H. dentatum using Self-Organizing Maps showing the effect of neighborhood shrinking to include the winning unit, i.e., when the vectors in the dataset reach the closest similarity. Figure S3. Codebbook vectors for H. caudiculatum and H. dentatum showing the clusters of differentially expresed genes with maximum neighbouring after training process. The codebook vectors represent the expression profile of genes associated to a given state after the constructionof the map [file 12870_2019_2182_MOESM3_ESM.zip › Additional information 3_FigS1.tif]

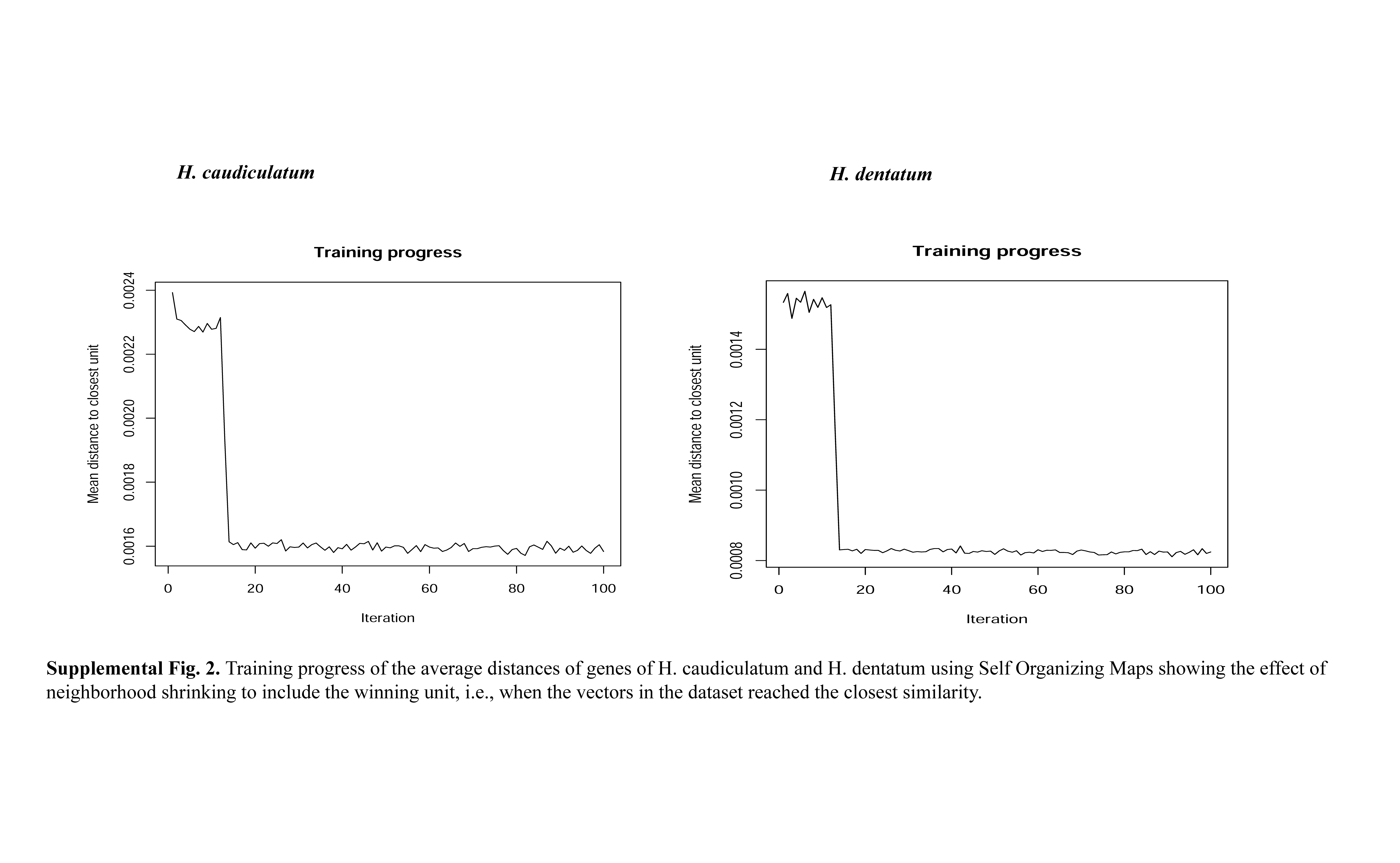

Supplement: Supplementary file 3 — Additional file 3 : Figure S1. Quality scores and accuracy of Illumina Hiseq 100-bp paired end reads for H. caudiculatum and H. dentatum after quality filters. Bad quality reads that showed values 20 on the histogram were discarded. All reads above the value of 30 indicates 99.9% of accuracy, regarding to bases correctly read by the sequencer, Figure S2. Training progress of the average distances of genes of H. caudiculatum and H. dentatum using Self-Organizing Maps showing the effect of neighborhood shrinking to include the winning unit, i.e., when the vectors in the dataset reach the closest similarity. Figure S3. Codebbook vectors for H. caudiculatum and H. dentatum showing the clusters of differentially expresed genes with maximum neighbouring after training process. The codebook vectors represent the expression profile of genes associated to a given state after the constructionof the map [file 12870_2019_2182_MOESM3_ESM.zip › Additional information 3_FigS2.tif]

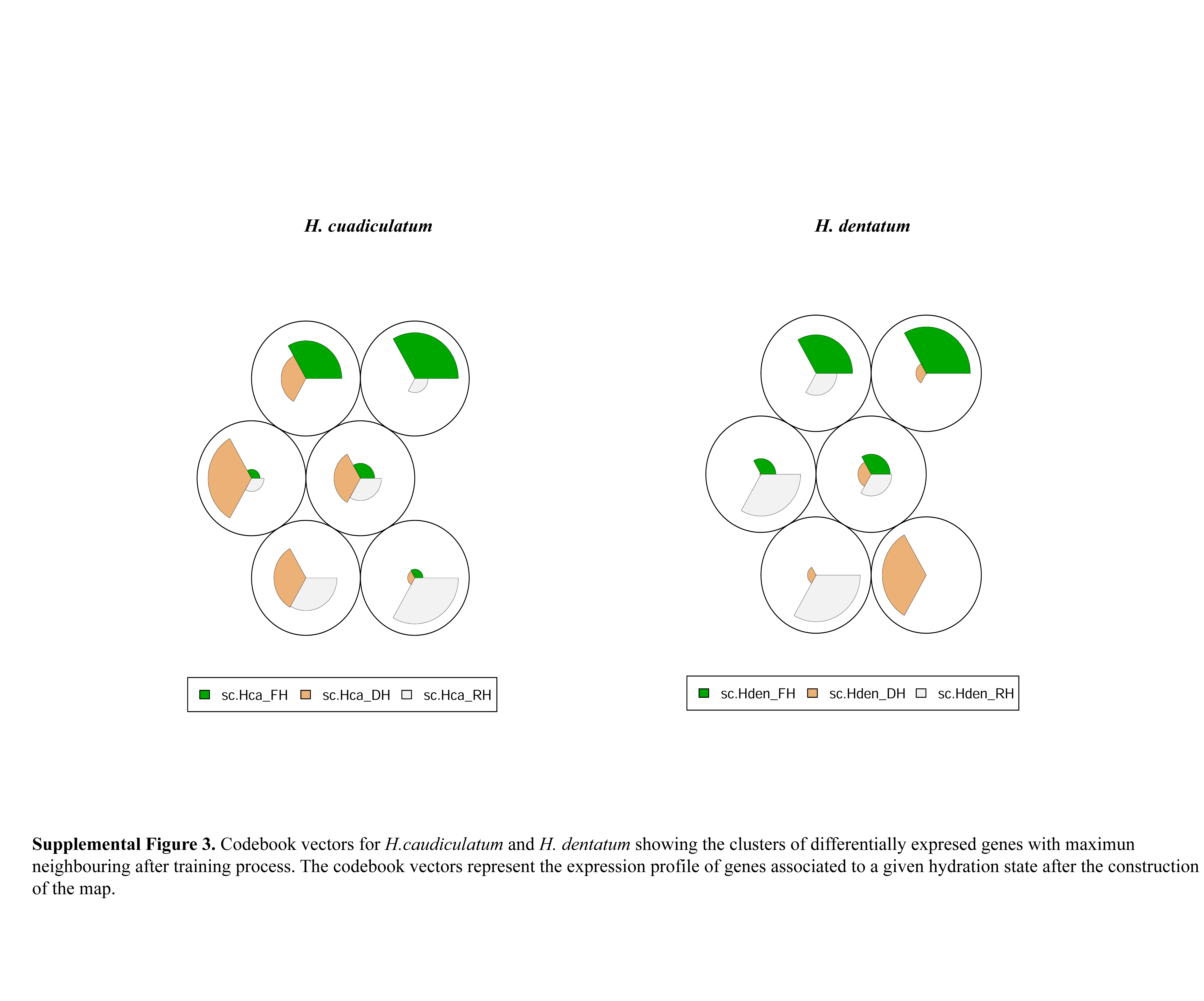

Supplement: Supplementary file 3 — Additional file 3 : Figure S1. Quality scores and accuracy of Illumina Hiseq 100-bp paired end reads for H. caudiculatum and H. dentatum after quality filters. Bad quality reads that showed values 20 on the histogram were discarded. All reads above the value of 30 indicates 99.9% of accuracy, regarding to bases correctly read by the sequencer, Figure S2. Training progress of the average distances of genes of H. caudiculatum and H. dentatum using Self-Organizing Maps showing the effect of neighborhood shrinking to include the winning unit, i.e., when the vectors in the dataset reach the closest similarity. Figure S3. Codebbook vectors for H. caudiculatum and H. dentatum showing the clusters of differentially expresed genes with maximum neighbouring after training process. The codebook vectors represent the expression profile of genes associated to a given state after the constructionof the map [file 12870_2019_2182_MOESM3_ESM.zip › Additional information 3_FigS3.tif]
